# Unsteady Aerodynamic Forces of Tandem Flapping Wings with Different Forewing Kinematics

**DOI:** 10.3390/biomimetics9090565

**Published:** 2024-09-19

**Authors:** Zengshuang Chen, Yuxin Xie, Xueguang Meng

**Affiliations:** State Key Laboratory for Strength and Vibration of Mechanical Structures, School of Aerospace Engineering, Xi’an Jiaotong University, Xi’an 710049, China; chenzengshuang@stu.xjtu.edu.cn (Z.C.); yxxie@stu.xjtu.edu.cn (Y.X.)

**Keywords:** tandem flapping wings, forewing kinematics, wing spacing, unsteady aerodynamic forces

## Abstract

Dragonflies can independently control the movement of their forewing and hindwing to achieve the desired flight. In comparison with previous studies that mostly considered the same kinematics of the fore- and hindwings, this paper focuses on the aerodynamic interference of three-dimensional tandem flapping wings when the forewing kinematics is different from that of the hindwing. The effects of flapping amplitude (*Φ*_1_), flapping mean angle ($\bar{\phi_{1}}$), and pitch rotation duration (Δ*tr*_1_) of the forewing, together with wing spacing (*L*) are examined numerically. The results show that *Φ*_1_ and $\bar{\phi_{1}}$ have a significant effect on the aerodynamic forces of the individual and tandem systems, but Δ*tr*_1_ has little effect. At a small *L*, a smaller *Φ*_1_, or larger $\bar{\phi_{1}}$ of the forewing can increase the overall aerodynamic force, but at a large *L*, smaller *Φ*_1_ or larger $\bar{\phi_{1}}$ can actually decrease the force. The flow field analysis shows that *Φ*_1_ and $\bar{\phi_{1}}$ primarily alter the extent of the impact of the previously revealed narrow channel effect, downwash effect, and wake capture effect, thereby affecting force generation. These findings may provide a direction for designing the performance of tandem flapping wing micro-air vehicles by controlling forewing kinematics.

## 1. Introduction

In the insect world, the dragonfly is unique because it has two pairs of tandem wings [1,2]. It can perform challenging flight maneuvers such as hovering, forward, and climbing flights by controlling the phase angle between the forewing and hindwing [3,4,5]. Dragonflies have always amazed us with their agility and flight pattern diversity and have motivated people to explore the unique aerodynamics of tandem wings. Understanding the physical mechanisms behind this can not only help biologists learn about dragonfly flight but can also provide guidance to engineers in designing new bionic tandem flapping wing micro-vehicles (FWMVs).

In order to provide a quick evaluation of the aerodynamic advantages of tandem wings, two-dimensional (2D) experimental and numerical simulation studies have become the preferred choice of researchers. Numerous studies have found that the distance and phase difference between the two wings are the key parameters affecting the performance of the forewing, hindwing, and whole system [6,7,8,9,10,11,12,13,14,15]. For example, Boschitsch et al. [8] experimentally found that the thrust and propulsive efficiency of the upstream foil was higher than that of an isolated foil only for relatively closely spaced foils, while the performance of the downstream foil depended strongly on the streamwise spacing and phase differentials between the foils. Zheng et al. [12] showed that tandem wings with in-phase outperformed a single wing in both hover and forward flight, while tandem wings with anti-phase were worse in both flights. This is because changes in the spacing and phase angle can affect the timing of the interaction between the vortex shed from the forewing and the hindwing, thus affecting the aerodynamic forces [6]. One study attributed this effect to two key wing–vortex interactions [13], i.e., the forewing leading edge vortex (LEV) induced a separation on the lower surface of the hindwing and the forewing trailing edge vortex (TEV) induced a separation on the upper surface of the hindwing. In another study, Joshi and Mysa [15] numerically examined the wake interaction with a downstream foil at larger streamwise spacings and proposed five favorable or unfavorable conditions for thrust generation. In addition to these studies, a few works have explored the effects of asymmetric pitch motion and stroke time asymmetry on the aerodynamic performance of tandem wings [16,17].

The feasibility was simple but limited by 2D studies, which prompted three-dimensional (3D) experimental and numerical simulations of tandem wings to be reported [18,19,20,21,22,23,24,25,26,27,28,29,30,31]. Hu and Deng [21] experimentally showed that tandem wings with in-phase flight enhanced the forewing lift by 17%, while the hindwing lift was reduced for most of the phase difference. Nagai et al. [24] indicated that the largest vertical force was generated in hovering and forward flight when tandem wings were in phase, while the vertical force decreased when the hindwing lagged the forewing. It was reported that the reduction in hindwing lift was due to the downwash generated by the forewing reducing the effective angle of attack, while the upwash generated by the LEV of the hindwing increased the forewing lift [21]. Recently, Meng et al. [26] demonstrated that the narrow channel effect between neighboring wings can increase the relative incoming velocity and similarly increase the forewing lift. In addition, some studies have focused on the differences between 2D and 3D tandem wings. Broering and Lian [28] pointed out that the LEV structure in the 3D case exhibits a spanwise variation compared with 2D results, which results in weaker vortex interactions between the forewing and hindwing. Arranz et al. [29] also showed that the 3D effect was detrimental to the vortex interactions between the wings, which caused the ratio between the average thrust of the hindwing and forewing to decrease from 80% in 2D to 70% in 3D. This suggests that a 3D investigation can provide more accurate forces and a more realistic flow compared with a 2D analysis.

In the above research, the kinematics of the forewing and hindwing are the same, except for the phase angle. These studies have emphasized that the aerodynamic forces of the hindwing depend strongly on the wake of the forewing. It is well known that the wake of a flapping wing is related to the morphological and kinematic parameters of the wing [32]. This has prompted researchers to explore the effect of different morphological and kinematics employed by the forewing and hindwing on the aerodynamic performance of tandem wings [33,34,35,36,37]. For instance, Chen et al. [34] demonstrated through 2D numerical simulation that the tandem wing hovering lift can be increased by modifying the flapping deviation to make the forewing below the hindwing. Jurado et al. [35] reported that the cycle-average thrust coefficient of the hindwing increased by 8% when the forewing aspect ratio was larger than that of the hindwing. Recently, the 2D numerical simulation results of Tiwari and Chandel [37] indicated that tandem wings hovering with anti-phase could increase lift by 46% when the stroke plane angle of the forewing was smaller than the hindwing. The literature also shows that aerodynamic interference affects the hindwing more than the forewing, and the aerodynamic behaviors of the hindwing determine the overall aerodynamic forces. The hindwing always works in the wake of the forewing, so the forewing kinematics largely dominate the magnitude of aerodynamic interference to which the hindwing is subjected and will affect the aerodynamic forces. However, there are relatively few 3D studies focusing on the effect of changes in forewing kinematics on the aerodynamic forces of tandem flapping wings in forward flight, which is the motivation for this work.

In this paper, the aerodynamic forces of 3D tandem flapping wings were numerically investigated at different forewing kinematics. The forewing kinematics parameters included flapping amplitude (*Φ*_1_), the flapping average angle ($\bar{\phi_{1}}$), and pitch rotation duration (Δ*tr*_1_). These are the key parameters that affect the aerodynamic forces and vortex structure of the flapping wing [38,39,40,41]. For example, Wu et al. [38] indicated that changing *Φ*_1_ and Δ*tr_1_* can effectively control the magnitude of the total aerodynamic force through numerical simulations. Chen et al. [39] demonstrated that in the early stage of the half-stroke, an advanced rotation can still increase lift when the wing outboard LEV breaks down. Other researchers experimentally measured the change in the wing kinematics of droneflies [40] and fruit flies [41] at different flight speeds. Their findings showed that the flapping amplitude first decreased and then increased [40,41]; the flapping average angle increased at lower speeds but changed little at speeds above 3 m/s [40]. This is the basis for considering these parameters. In addition, the coupling effects of these parameters and wing spacing (*L*) were examined. The law of influence of these parameters on the aerodynamic forces was obtained, and the flow mechanism behind this was revealed by flow analysis. The remainder of this study is organized as follows: In Section 2, the tandem wings model and kinematic definition are presented, and the numerical methodology is illustrated and verified. The results of the effects of different parameters are shown and explained in detail in Section 3. Finally, conclusions are summarized in Section 4.

## 2. Materials and Methods

### 2.1. Geometry and Kinematics

**Figure 1:**
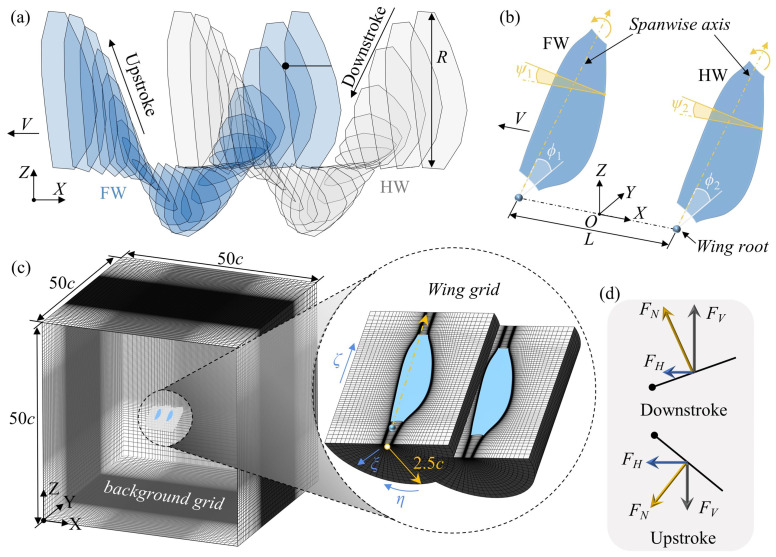
Schematic: (**a**) the forward motion of tandem flapping wings, (**b**) the coordinate system and definition of flapping wing motion, (**c**) the computational grid, and (**d**) the definition of the projection of aerodynamic force.

Two wings in an in-line tandem arrangement perform vertical flapping and pitch changes while simultaneously moving forward at a certain speed (*V*). A schematic diagram of the motion trajectory is shown in Figure 1a. The models of the forewing (FW) and hindwing (HW) are in the form of a flat plate with rounded leading and trailing edges and a thickness of 0.03*c* (*c* is the mean chord length). The fore- and hindwings are the same shape, and the planform of the wing is similar to that of the wing of *Eristalis tenax* [40]. The aspect ratio (*R*/*c*) and the radius of the second moment (*r*_2_) of the wing are 3.75 and 0.56*R* (*R* is the wing length), respectively.

The reference coordinate system and a sketch of the wing in flapping motion are shown in Figure 1b. The vertical flapping and pitching motions of the wing can be decomposed into two key kinematic angles around the *X*-axis and the spanwise axis, i.e., the flapping angle (*ϕ*) and the pitching angle (*ψ*). *ϕ* is defined as the angle between the spanwise axis and the *Y*-axis, and *ψ* is defined as the angle between the wing surface plane and the horizontal plane (XOY plane). The flapping angle (*ϕ*) and the pitching angle (*ψ*) in the present study follow our previous works [42] and are described by the following cosine and trapezoidal functions, respectively:

(1) $$\phi_{i}(t)=0.5\Phi_{i}\cos(2\pi \mathrm{ft})+\bar{\phi_{i}},$$

(2) $$\psi_{i}(t)=\left\{\begin{array}{ll} \alpha_{u}-\frac{\Psi }{\Delta t_{ri}}\left\{(t-t_{1})-\frac{\Delta t_{ri}}{2\pi }\sin\left[\frac{2\pi (t-t_{1})}{\Delta t_{ri}}\right]\right\}, & 0\leq t<\frac{\Delta t_{ri}}{2}\\ \alpha_{d}, & \frac{\Delta t_{ri}}{2}\leq t<\frac{T}{2}-\frac{\Delta t_{ri}}{2}\\ \alpha_{d}+\frac{\Psi }{\Delta t_{ri}}\left\{(t-t_{2})-\frac{\Delta t_{ri}}{2\pi }\sin\left[\frac{2\pi (t-t_{2})}{\Delta t_{ri}}\right]\right\}, & \frac{T}{2}-\frac{\Delta t_{ri}}{2}\leq t<\frac{T}{2}+\frac{\Delta t_{ri}}{2}\\ \alpha_{u}, & \frac{T}{2}+\frac{\Delta t_{ri}}{2}\leq t<T-\frac{\Delta t_{ri}}{2}\\ \alpha_{u}-\frac{\Psi }{\Delta t_{ri}}\left\{(t-t_{3})-\frac{\Delta t_{ri}}{2\pi }\sin\left[\frac{2\pi (t-t_{3})}{\Delta t_{ri}}\right]\right\}, & T-\frac{\Delta t_{ri}}{2}\leq t<T \end{array}\right.$$

where the subscript *i* denotes the wing (*i* = 1 FW, *i* = 2 HW). In Equation (1), *Φ_i_* is the flapping amplitude, *t* is the time, *f* is the flapping frequency, and $\bar{\phi_{i}}$ is the average flapping angle. In Equation (2), Ψ is the pitching amplitude, and *α_u_* and *α_d_* are the angles of attack in the middle of the upstroke and downstroke, respectively. Moreover, *t*_1_ = −Δ*tr*_i_/2, *t*_2_ = (*T* − Δ*tr*_i_)/2, and *t*_3_ = *T* − Δ*tr*_i_/2, where Δ*tr*_i_ is the duration time of the pitching rotation and *T* is the period of a flapping cycle. Referring to the experimentally measured data on the forward flight of the dronefly [40], *Φ*_2_ = 100°, $\bar{\phi_{2}}$ = 25°, and Δ*tr*_2_ = 0.3*T*. The advance ratio (*J*) is often used to denote the flight speed of insects and is defined as the ratio of flight speed to wingtip speed (*J* = *V*/2*ΦfR*) [41]. Using the wingtip speed of the hindwing as a standard, *J* = *V*/2*Φ*_2_*fR* = 0.4, which corresponds to the medium forward flight speed of the dronefly [40]. In addition, *α_u_* = 50° and *α_d_* = −15°, which ensures that the upward vertical force and forward horizontal force can be generated at this *J*.

In tandem, the FW and HW root spacing in the X-direction is defined as *L* (Figure 1b). In this paper, the kinematics of the HW were always kept constant; only that of the FW are modified, and the period of flapping cycle of the FW was always the same as that of the HW. The FW kinematics parameters considered were flapping amplitude (*Φ*_1_), flapping mean angle ($\bar{\phi_{1}}$), and pitch rotation duration (Δ*tr*_1_). The specific parameters to be modified are shown in Table 1. In addition, the coupling effects of these parameters with *L* were considered, and *L* took values in the range 1.5*c*–5*c* (Table 1).

### 2.2. Computational Method and Validation

As the motion of the wing is unsteady, and the mean flapping speed and forward flight speed of the wing are very low, the governing equations for the flow of tandem flapping wings are the three-dimensional incompressible unsteady Navier–Stokes equations. The mean chord length (*c*), the mean flapping velocity at *r*_2_ (*U_r_* = 2*Φfr*_2_), and *c*/*U_r_* are used as reference length, velocity, and time, respectively, to nondimensionalize the Navier–Stokes equations. The dimensionless forms of the equations are as follows:

(3) ∇·u=0,∂u/∂t*+(u·∇)u+∇p−∇2u/Re=0,

where ***u*** and *p* represent the dimensionless fluid velocity and pressure, respectively. *Re* denotes the Reynolds number and is calculated by *cU_r_*/*v*, where *v* is the kinematic viscosity of the fluid. The governing equations are solved numerically using a finite-difference-based in-house solver. Based on the artificial compressibility method [43,44], the in-house solver couples the pressure and velocity fields by introducing a pseudo-time derivative of pressure and an artificial compressibility constant in the continuity equation. The time derivative term of the momentum equation is discretized by a second-order, three-point, backward difference scheme. For the viscous term, a second-order central difference scheme is used, and for the convective term, an upwind difference scheme based on the vector flux-difference splitting method is employed. Since tandem flapping wings move at low *Re* (=200), the flow generated by the wings presents mainly laminar structure and thus no turbulence model is considered in the in-house solver. For more information on the solver, please see our previous work [26,27].

As shown in Figure 1c, the grid that discretizes the computational domain contains the wing grid and the background grid. For each wing grid, there is a body-fit O-H grid that extends 2.5*c* from the wing surface and about 1c at the extension near the wing root and tip, and *ξ*, *η*, and *ζ* denote the normal, around the wing, and spanwise directions, respectively. In addition, a background Cartesian grid extends to the far-field boundary of the domain. The outer boundary of the background grid is 20*c* and 30*c* from the wing root of FW in the negative and positive X directions, respectively, and at 25*c* in both the Z and Y directions. For far-field boundary conditions, at the inflow boundary, the velocity component is specified by the relative velocity at the boundary, and the pressure is interpolated externally by the interior point. At the outflow boundary, the pressure is set to be the static pressure of the stationary air, and the velocity is interpolated externally from the interior. At the wing surface, an impermeable no-slip boundary condition is used, and the pressure at the boundary is obtained from the normal component of the momentum equation in the moving coordinate system.

**Figure 2:**
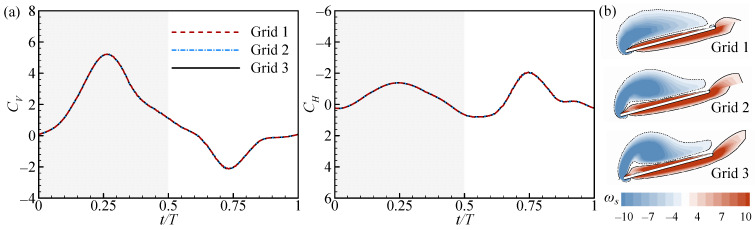
(**a**) Instantaneous aerodynamic force coefficients of the hindwing for three grids. (**b**) The spanwise vorticity contours at the *r*_2_ section of the hindwing in mid-downstroke.

Grid density validation was performed before numerical computation, and three grids were considered. The kinematic parameters of the forewing were set to *Φ*_1_ = 100°, $\bar{\phi_{1}}$ = 25°, and Δ*tr*_1_ = 0.3*T*, and the wing spacing (*L*) was fixed to 4*c*. For grid 1, the dimensions of the wing grid were 25 × 45 × 37 in the *ξ*, *η*, and *ζ* directions, respectively (the first grid layer thickness was 0.002*c*), and the dimensions of the background grid were 62 × 62 × 62 in the X, Y, and Z directions, respectively. For grids 2 and 3, the corresponding grid dimensions were 37 × 67 × 55 (0.0015*c*) and 93 × 93 × 93 and 55 × 99 × 83 (0.001*c*) and 140 × 140 × 140, respectively. The tandem flapping wings began to flap in a quiescent fluid, and after about four cycles, the flow became periodic. A dimensionless time (*t*/*T*) was used to represent the variations between 0 and 1 for each flapping period, where the downstroke was in the range 0–0.5 and the upstroke in the range 0.5–1. Figure 2a plots the aerodynamic force coefficients of the HW for different grids. It can be seen that the aerodynamic forces calculated for the three grids almost coincide. However, the spanwise vorticity at the *r*_2_ section of the HW becomes more exquisite with the encryption of the grid (see Figure 2b). Then, the effects of domain size and time step were also verified accordingly. Eventually, grid 3 and time step 0.02 were used for the calculations of the current study.

**Figure 3:**
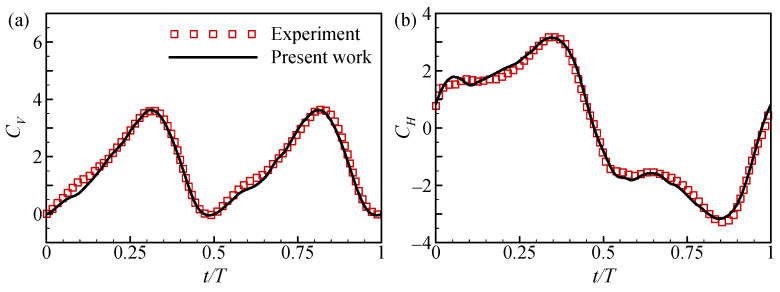
Comparison of (**a**) vertical and (**b**) horizontal force coefficients obtained from numerical calculations with experimental data.

The accuracy of the numerical method was verified in our previous studies, and it agrees well with both the experimental results for a single flapping wing and the numerical results for tandem wings [26,42]. To further test the correctness of the in-house solver, the total instantaneous vertical and horizontal force coefficients for tandem flapping wings hovering in-phase were compared with the experimentally measured values by Nagai et al. [24], as shown in Figure 3. It is evident that the calculated force coefficients are in good agreement with the measured data.

### 2.3. Data Analysis

The projections of the aerodynamic forces (*F_N_*) acting on the wing in the vertical and horizontal directions are denoted as *F_V_* and *F_H_*, respectively, as shown in Figure 1d. Since the wing flaps vertically, during a flapping cycle, *F_V_* points in the positive and negative directions of the *Z*-axis in the downstroke and upstroke, respectively, while *F_H_* always points in the negative direction of the *X*-axis. The positive vertical and horizontal forces are defined in the positive direction of the *Z*-axis and *X*-axis, respectively. The dimensionless vertical and horizontal force coefficients are defined as follows:

(4) $$C_{\mathrm{Vi}}=2F_{\mathrm{Vi}}/\rho U_{r}^{2}S,$$

(5) $$C_{\mathrm{Hi}}=2F_{\mathrm{Hi}}/\rho U_{r}^{2}S,$$

where the subscript *i* denotes the wing (*i* = 1 FW, *i* = 2 HW), *S* is the area of one wing, and *ρ* is the air density. The instantaneous *C_Vi_* and *C_Hi_* were integrated to obtain cycle-averaged vertical and horizontal force coefficients ($\bar{C_{\mathrm{Vi}}}$ and $\bar{C_{\mathrm{Hi}}}$), respectively. In the current study, these averages were calculated from the instantaneous coefficients of a stable flapping cycle (after about four cycles, the flow became periodic).

The pressure coefficients are defined as follows:

(6) $$C_{p}=2(p-p_{\infty })/\rho U_{r}^{2},$$

where *p* is the calculated pressure and *p*_∞_ is the pressure at infinity.

## 3. Results

### 3.1. Effect of Φ_1_

In order to perform a better analysis, the increments of $\bar{C_{V}}$ and $\bar{C_{H}}$ of the FW and the HW relative to a single wing (SW) were calculated at different parameters, denoted as ∆$\bar{C_{\mathrm{Vi}}}$ and ∆$\bar{C_{Hi}}$ (*i* = 1 FW, *i* = 2 HW), respectively. The increment of $\bar{C_{V}}$ and $\bar{C_{H}}$ of the total tandem flapping wings (denoted as ∆$\bar{C_{VT}}$ and ∆$\bar{C_{\mathrm{HT}}}$) is equal to the sum of the corresponding increments of FW and HW. For detailed $\bar{C_{V}}$ and $\bar{C_{H}}$ values for a single flapping wing at different parameters, please see Appendix A.

**Figure 4:**
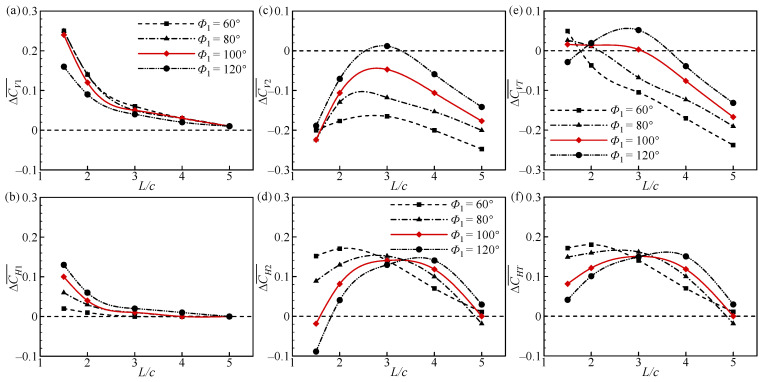
Increments of $\bar{C_{V}}$ and $\bar{C_{H}}$ of the (**a**,**b**) forewing, (**c**,**d**) hindwing, and (**e**,**f**) total tandem flapping wings relative to a single flapping wing at different *Φ*_1_ and *L* values.

Figure 4 plots the increments of $\bar{C_{V}}$ and $\bar{C_{H}}$ for the forewing, hindwing, and overall tandem flapping wings relative to a single flapping wing at different *Φ*_1_ and *L* values. In Figure 4a,b, the aerodynamic forces of the FW are maximum at *L* = 1.5*c*, and there is a monotonically decreasing trend with the increase in *L*. At *L* ≥ 4*c*, the aerodynamic forces of the FW decrease to the same as those of the single wing. In addition, the effect of *Φ*_1_ is small at *L* > 2*c*. In contrast, at *L* ≤ 2*c*, the effect of *Φ*_1_ is significant, with ∆$\bar{C_{V1}}$ decreasing and ∆$\bar{C_{H1}}$ increasing as *Φ*_1_ increases. This indicates that at small spacing (*L* ≤ 2*c*), *Φ*_1_ > *Φ*_2_ (100°) is more conducive to increasing the horizontal thrust of the FW, and *Φ*_1_ ˂ *Φ*_2_ (100°) is more conducive to increasing the vertical lift of the FW.

In Figure 4c,d, the aerodynamic changes in the HW are different from those of the FW. The aerodynamic forces of the HW first increase and then decrease with the increase in *L*, and the effect of *Φ*_1_ is particularly remarkable. It is worth noting that in the majority of cases, ∆$\bar{C_{V2}}$ < 0 and ∆$\bar{C_{H2}}$ > 0, which indicates that an increase in horizontal force is accompanied by a decrease in vertical force. At *L* = 3*c*, ∆$\bar{C_{V2}}$ is maximum at *Φ*_1_ = 120° and decreases gradually with the decrease in *Φ*_1_, while ∆$\bar{C_{H2}}$ changes very little. At a small spacing (*L* = 1.5*c* and 2*c*), ∆$\bar{C_{H2}}$ decreases as *Φ*_1_ increases. However, at a large spacing (*L* = 4*c*), ∆$\bar{C_{H2}}$ increases as *Φ*_1_ increases. This suggests that at small spacing, it is more favorable to increase the horizontal thrust of the HW when *Φ*_1_ ˂ *Φ*_2_ (100°), while at large spacing, it is more favorable to increase the horizontal thrust of the HW when *Φ*_1_ > *Φ*_2_ (100°).

In Figure 4e,f, it can be seen that ∆$\bar{C_{VT}}$ < 0 and ∆$\bar{C_{\mathrm{HT}}}$ > 0 for most cases, which is similar to the variation in the HW. This demonstrates that the aerodynamic forces of the HW have a dominant contribution to the overall aerodynamic forces. At *L* = 1.5*c*, ∆$\bar{C_{VT}}$ decreases with increasing *Φ*_1_, while it increases with increasing *Φ*_1_ at *L* ≥ 2*c*. The change in ∆$\bar{C_{\mathrm{HT}}}$ at different *Φ*_1_ and *L* values is consistent with that of the HW. At a small spacing, ∆$\bar{C_{VT}}$ and ∆$\bar{C_{\mathrm{HT}}}$ are maximized at *Φ*_1_ = 60°; at a large spacing, they are maximized at *Φ*_1_ = 120°. This suggests that tandem flapping wings can be used with different forewing flapping amplitudes to maximize the aerodynamic increment at different wing spacings. It also provides engineers with evidence that a good combination of flapping amplitude and wing spacing can boost aerodynamic forces in the design of tandem flapping wing micro air vehicles.

**Figure 5:**
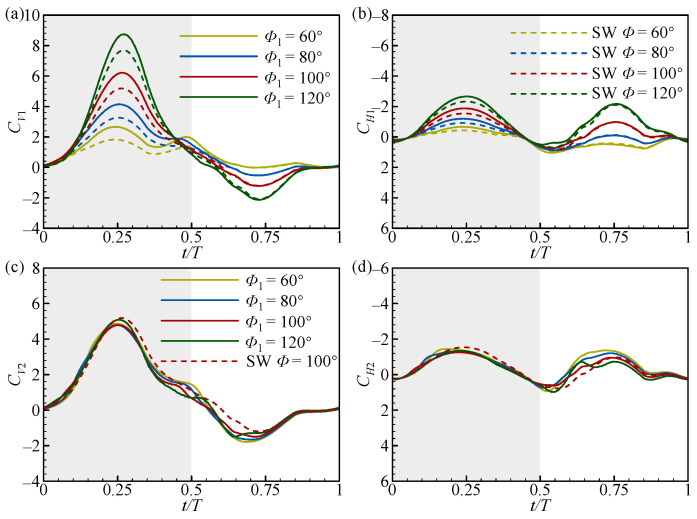
The transient vertical and horizontal force coefficients of (**a**,**b**) the FW and (**c**,**d**) the HW with *L* = 1.5*c* at different *Φ*_1_ values. The dashed lines in the figures represent the results for the SW. The grey and white areas indicate the downstroke and upstroke, respectively.

**Figure 6:**
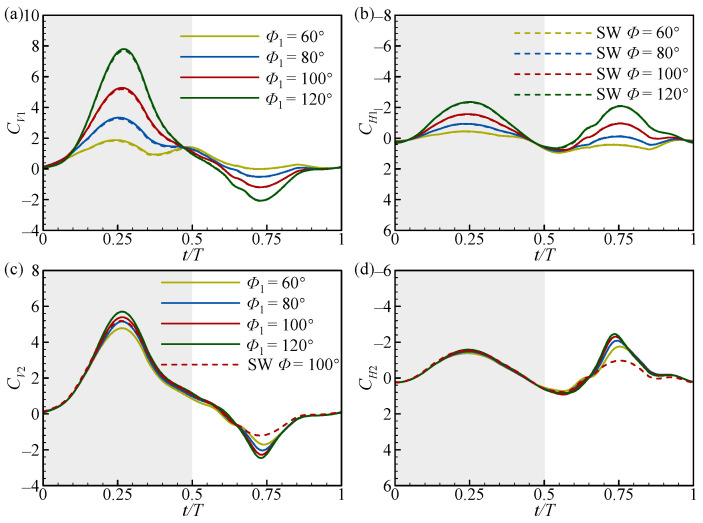
The transient vertical and horizontal force coefficients of (**a**,**b**) the FW and (**c**,**d**) the HW with *L* = 4*c* at different *Φ*_1_ values. The dashed lines in the figures represent the results for the SW. The grey and white areas indicate the downstroke and upstroke, respectively.

To further explain the variation in the increments in cycle-averaged aerodynamic forces with *Φ*_1_, the transient force coefficient curves of the FW and the HW at two typical spacings (*L* = 1.5*c* and 4*c*) are plotted in Figure 5 and Figure 6, together with those of the SW (dashed curves). As can be seen in Figure 5a,b, in the downstroke (*t*/*T* ≈ 0.25), the positive *C_V_*_1_ and *C_H_*_1_ peaks gradually increase as *Φ*_1_ increases and are both higher than that of the SW. In the upstroke (*t*/*T* ≈ 0.75), the negative *C_V_*_1_ and positive *C_H_*_1_ peaks also increase as *Φ*_1_ increases, but almost overlap with the results of the SW. This is the reason that the change in ∆$\bar{C_{V1}}$ and ∆$\bar{C_{H1}}$ as *Φ*_1_ increases is opposite (Figure 4a,b). This also shows that the wing–wing interaction can enhance the aerodynamic forces of the FW in the downstroke at a small spacing (*L* = 1.5*c*). However, at a large spacing (*L* = 4*c*), the force curves of the FW completely overlap with those of the SW, as shown in Figure 6a,b. This indicates that the wing–wing interaction has no aerodynamic effect on the FW in this case.

In Figure 5c,d, in the downstroke (*t*/*T* ≈ 0.25), the positive *C_V_*_2_ and *C_H_*_2_ peaks are lower than those of the SW and change little as *Φ*_1_ increases, where the *C_V_*_2_ is slightly higher than the other cases at *Φ*_1_ = 120°. In the upstroke (*t*/*T* ≈ 0.75), the negative *C_V_*_2_ and positive *C_H_*_2_ peaks decrease as *Φ*_1_ increases, where the *C_H_*_2_ peak decreases to below that of the SW at *Φ*_1_ = 120°. This leads to a little change in ∆$\bar{C_{V2}}$ and a gradual decrease in ∆$\bar{C_{H2}}$ as *Φ*_1_ increases at *L* = 1.5*c* (Figure 4c,d). When *L* = 4*c*, in the downstroke (*t*/*T* ≈ 0.25), the positive *C_V_*_2_ peak increases as *Φ*_1_ increases, and the positive *C_V_*_2_ peak is consistent with that of the SW at *Φ*_1_ = 80°, but the positive *C_H2_* peak is almost the same as that of the SW for all *Φ*_1_ (Figure 6c,d). In the upstroke (*t*/*T* ≈ 0.75), the negative *C_V2_* and positive *C_H2_* peaks are both higher than the SW and increase as *Φ*_1_ increases (Figure 6c,d). This results in ∆$\bar{C_{V2}}$ and ∆$\bar{C_{H2}}$ increasing as *Φ*_1_ increases at a large spacing (*L* = 4*c*). Note that the variation in the aerodynamic forces for the HW in the upstroke as *Φ*_1_ increases in the large spacing case is the opposite of the small spacing case (Figure 5d and Figure 6d). This also suggests that the aerodynamic effect of *Φ*_1_ in the upstroke is different at different *L* values.

**Figure 7:**
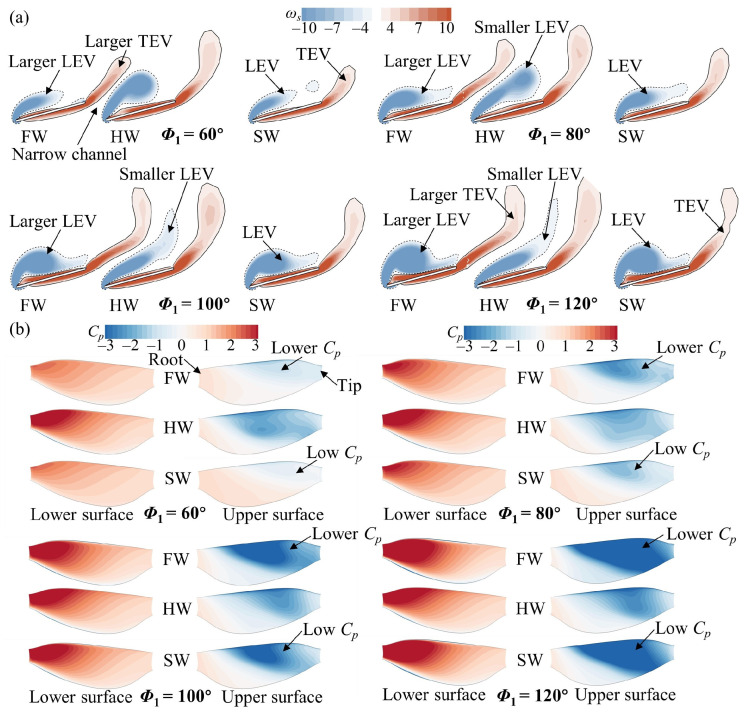
(**a**) Spanwise vorticity (*ω_s_*) at the *r*_2_ plane and (**b**) wing surface pressure distribution of the FW, the HW, and the SW for different *Φ*_1_ values at *L* = 1.5*c* and *t*/*T* = 0.25.

A detailed elucidation of the effects of wing–wing interactions at different *Φ*_1_ and *L* values (1.5*c* and 4*c*) is given below. The flow structure is first analyzed at *L* = 1.5*c*. The spanwise vorticity and wing pressure distribution of the FW, the HW, and the SW for different *Φ*_1_ values at *t*/*T* = 0.25 are plotted in Figure 7. An increase in *Φ*_1_ means that the flapping velocity becomes larger. In Figure 7a, the leading-edge vortex (LEV) and trailing-edge vortex (TEV) generated by the SW gradually become larger as *Φ*_1_ increases. The corresponding negative pressure (suction force) on the upper wing surface also increases (Figure 7b), leading to a gradual increase in aerodynamic forces (Figure 5a,b). Similarly, the LEV and the negative pressure on the upper wing surface of the FW become larger as *Φ*_1_ increases, surpassing those of the SW (Figure 7a,b). This is attributed to the narrow channel effect between neighboring wings [26,45], which increases the incoming flow velocity relative to the wing, thereby producing a larger LEV. Consequently, the FW exhibits superior aerodynamic forces compared with the SW at different *Φ*_1_ values (Figure 5a,b). Although the narrow channel effect also increases the relative incoming velocity of the HW, it should be noted that the TEV of the FW is always above the HW (Figure 7a). Thus, the HW is affected by the downwash induced by the wake of the FW [45], resulting in both a smaller LEV and less negative pressure on the upper wing surface compared with the SW (Figure 7). Therefore, the aerodynamic forces of the HW are lower than those of the SW at different *Φ*_1_ values (Figure 5c,d).

**Figure 8:**
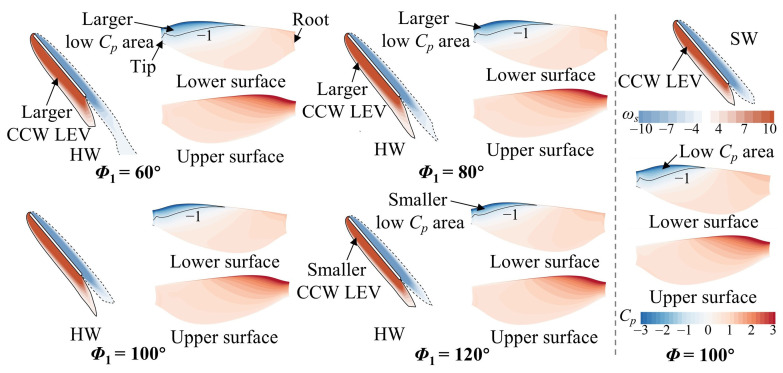
Spanwise vorticity (*ω_s_*) at the *r*_2_ plane and wing surface pressure distribution of the HW for different *Φ*_1_ values at *L* = 1.5*c* and *t*/*T* = 0.75. The results for the SW at *Φ* = 100° are also plotted in the figure.

**Figure 9:**
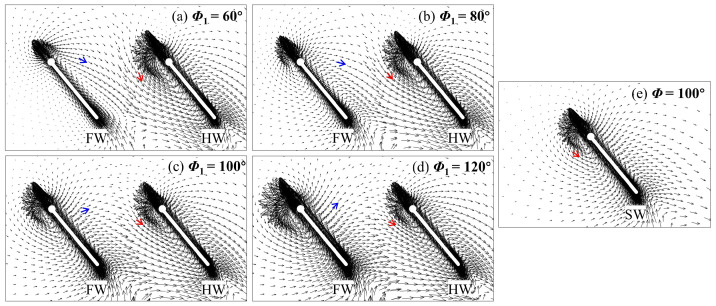
Velocity vectors at the *r*_2_ plane of the FW and the HW for different (**a**–**d**) *Φ*_1_ values at *L* = 1.5*c* and *t*/*T* = 0.75. (**e**) The result for the SW at *Φ* = 100° is also plotted in the figure.

In the upstroke, the main focus is on the flow variations in the HW. In Figure 8, the counterclockwise (CCW) LEV and wing surface pressure of the HW are observed to be larger at *Φ*_1_ = 60° and 80° and smaller at *Φ*_1_ = 120° compared with those of the SW. At a small spacing (*L* = 1.5*c*), the HW cannot take advantage of the wake capture effect to form a larger CCW LEV because the wake of the FW already developed downstream [45]. To further elucidate the reason, the velocity vectors at the *r*_2_ section are plotted in Figure 9. It can be observed that the fluid above the upper wing surface of the FW is pushed away, and the velocity vector direction (blue arrows) gradually deflects counterclockwise as *Φ*_1_ increases, which results in the velocity vector direction (red arrows) of the lower wing surface of the HW gradually deflecting counterclockwise as well. This is because a change in *Φ*_1_ implies a difference in flapping velocity, which results in a discrepancy in the ability to perturb the fluid. Compared with the SW, the velocity vector direction (red arrows) of the lower wing surface of the HW is more vertical at *Φ*_1_ ˂ *Φ*_2_ (100°) and more horizontal at *Φ*_1_ > *Φ*_2_ (100°). Correspondingly, the CCW LEV of the is larger than that of the SW at *Φ*_1_ = 60° and 80° and smaller at *Φ*_1_ = 120° (Figure 8). This leads to changes in the aerodynamic forces of the HW (Figure 5c,d).

**Figure 10:**
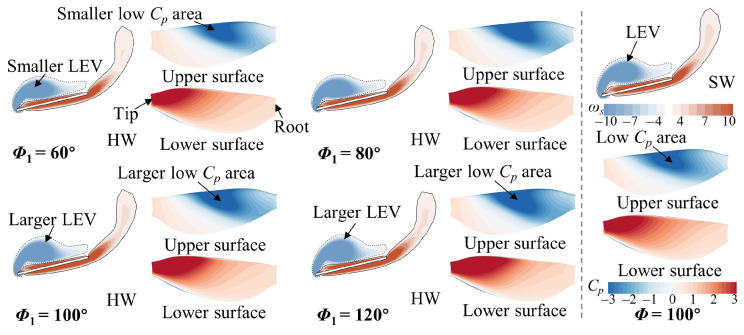
Spanwise vorticity (*ω_s_*) at the *r*_2_ plane and wing surface pressure distribution of the HW for different *Φ*_1_ values at *L* = 4*c* and *t*/*T* = 0.25. The results for the SW at *Φ* = 100° are also plotted in the figure.

**Figure 11:**
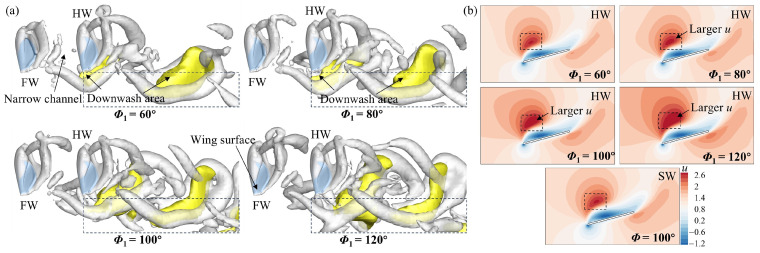
(**a**) Iso-Q surface (Q = 0.1, gray) showing the vortex structures and iso-vertical velocity (*v*) surface (*v* = −0.45, yellow) showing the downwash and (**b**) velocity (*u*) contour at the *r*_2_ plane of the HW for different *Φ*_1_ values at *L* = 4*c* and *t*/*T* = 0.25. *u* is the velocity vector component that is parallel to the stroke plane and perpendicular to the wingspan.

Then, the case of *L* = 4*c* was analyzed, primarily examining the flow structure of the HW. In Figure 10, in the downstroke (*t*/*T* = 0.25), the LEV and low *C_p_* area on the upper wing surface of the HW are larger at *Φ*_1_ = 100° and 120° and smaller at *Φ*_1_ = 60° compared with those of the SW. The underlying flow reason for this can be found in Figure 11. In Figure 11b, the relative incoming velocity of the HW increases as *Φ*_1_ increases, indicating that the narrow channel effect is strengthened as *Φ*_1_ increases. This leads to the lift of the HW becoming larger with the increase in *Φ*_1_ (Figure 6c). It is notable that the relative incoming velocity of the HW is larger at *Φ*_1_ = 80°, 100°, and 120° compared with that of the SW, whereas it is almost the same at *Φ*_1_ = 60°. Additionally, the HW is affected by the downwash effect accompanying the wake in the downstroke when it is far enough away from the FW [45]. In Figure 11a, it can be noticed that at *Φ*_1_ = 60° and 80°, the downwash area is located below the HW. However, as *Φ*_1_ increases (100° and 120°), the downwash area gradually moves backward to be located behind the HW. As a result, at *Φ*_1_ = 60°, the lift of the HW is lower than that of the SW because of the downwash effect; at *Φ*_1_ = 80°, the narrow channel effect and downwash effect are in competition, resulting in the lift of the HW being almost the same as that of the SW; at *Φ*_1_ = 100° and 120°, the narrow channel effect is enhanced and the downwash effect is diminished, so the lift of the HW is larger than that of the SW (Figure 6c).

**Figure 12:**
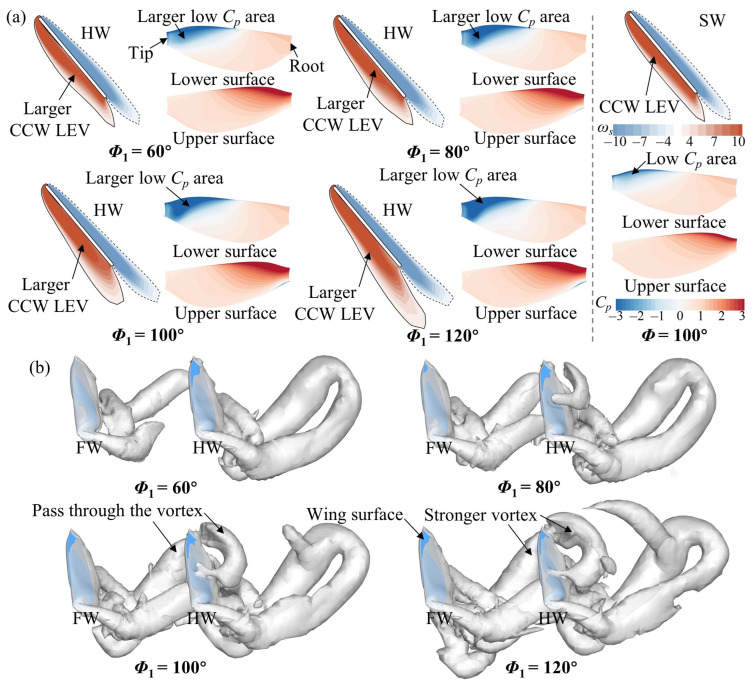
(**a**) Spanwise vorticity (*ω_s_*) at the *r*_2_ plane and wing surface pressure distribution of the HW, and (**b**) iso-Q surface (Q = 0.5) of the FW and the HW for different *Φ*_1_ values at *L* = 4*c* and *t*/*T* = 0.75. The results for the SW at *Φ* = 100° are also plotted in (**a**).

Figure 12 plots the vortex structure and wing surface pressure of the HW for different *Φ*_1_ values in the upstroke (*t*/*T* = 0.75). In Figure 12a, the CCW LEV and low *C_p_* area on the lower surface of the HW become larger and surpass those of the SW as *Φ*_1_ increases. This leads to a variation in the aerodynamic force of the HW (Figure 6d). This is because the hindwing can capture the wake of the forewing during the upstroke, utilizing the wake-induced downwash to increase aerodynamic forces [26]. In Figure 12b, the HW passes through the vortex of the FW, thus increasing the aerodynamic force (Figure 6d). Moreover, the vortex of the FW becomes stronger as *Φ*_1_ increases (Figure 12b), implying that the wake capture effect can be strengthened. The reasons for the aerodynamic changes in the HW in the upstroke are different compared with the small spacing case.

From the above analysis, it can be concluded that the effect of *Φ*_1_ on the aerodynamic performance and flow structure of the tandem flapping wings is significant, and the aerodynamic effect of *Φ*_1_ is different at different *L* values. This is because the change in *Φ*_1_ alters the impact degree of the previously proposed narrow channel effect, the downwash effect, and the wake capture effect.

### 3.2. Effect of $\bar{\phi_{1}}$

**Figure 13:**
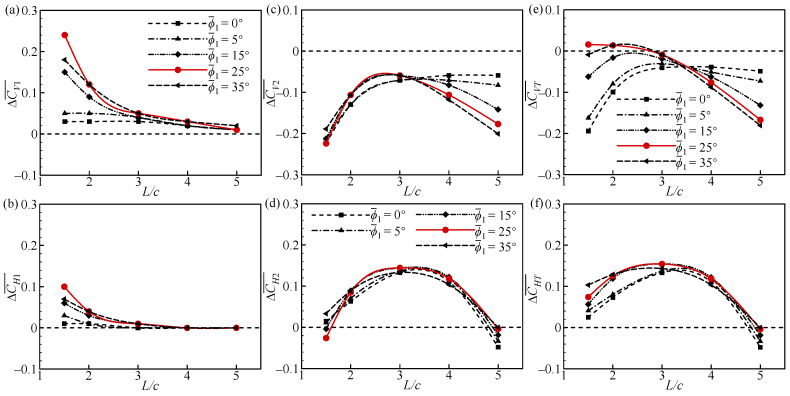
Increments of $\bar{C_{V}}$ and $\bar{C_{H}}$ of the (**a**,**b**) forewing, (**c**,**d**) hindwing, and (**e**,**f**) total tandem flapping wings relative to a single flapping wing at different $\bar{\phi_{1}}$ and *L* values.

Figure 13 shows the increments of $\bar{C_{V}}$ and $\bar{C_{H}}$ for the forewing, hindwing, and overall tandem flapping wings relative to a single wing at different $\bar{\phi_{1}}$ and *L* values. In Figure 13a,b, the aerodynamic forces of the FW decrease as *L* increases, and the effect of $\bar{\phi_{1}}$ is more significant at *L* ≤ 2*c*. At *L* = 1.5*c*, ∆$\bar{C_{V1}}$ and ∆$\bar{C_{H1}}$ first increase and then decrease as $\bar{\phi_{1}}$ increases, obtaining a maximum at $\bar{\phi_{1}}$ = 25°. This indicates that the maximum aerodynamic force increment of the FW can be obtained when the FW and the RW are in the same initial position.

In Figure 13c,d, the aerodynamic forces of the HW tend to increase and then decrease as *L* increases, and the effect of $\bar{\phi_{1}}$ is particularly significant for the vertical force at *L* ≥ 4*c*. At *L* = 5*c*, ∆$\bar{C_{V2}}$ < 0 and decreases as $\bar{\phi_{1}}$ increases (Figure 13c). The effect of $\bar{\phi_{1}}$ on ∆$\bar{C_{H2}}$ is relatively small at different *L* values. This leads to a significant effect of $\bar{\phi_{1}}$ on ∆$\bar{C_{VT}}$ at *L* ≤ 2*c* and *L* ≥ 4*c*, and a smaller effect on ∆$\bar{C_{\mathrm{HT}}}$ (Figure 13e,f). At *L* = 1.5*c* and 5*c*, ∆$\bar{C_{VT}}$ achieves its maximum value at $\bar{\phi_{1}}$ = 25° and 0°, respectively. This demonstrates that the tandem flapping wings can achieve the maximum vertical force increment at different wing spacings by modifying the flapping mean angle of the forewing. This also suggests that a good combination of flapping mean angle and wing spacing is critical in the takeoff design of tandem flapping wing micro-air vehicles.

**Figure 14:**
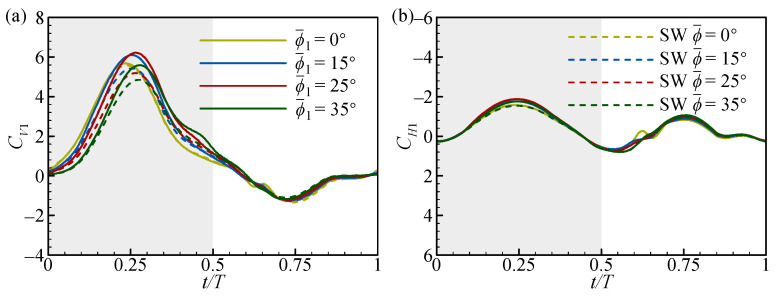
The transient (**a**) vertical and (**b**) horizontal force coefficients of the FW with *L* = 1.5*c* at different $\bar{\phi_{1}}$ values. The dashed lines in the figures represent the results for the SW. The grey and white areas indicate the downstroke and upstroke, respectively.

**Figure 15:**
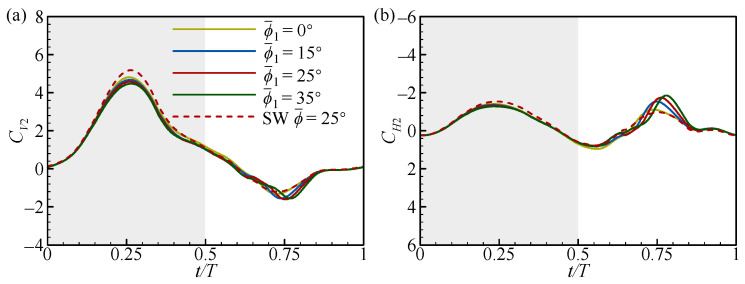
The transient (**a**) vertical and (**b**) horizontal force coefficients of the FW with *L* = 5*c* at different $\bar{\phi_{1}}$ values. The dashed lines in the figures represent the results for the SW. The grey and white areas indicate the downstroke and upstroke, respectively.

At *L* = 1.5*c* and 5*c*, the transient forces of the FW (Figure 14) and the RW (Figure 15) for different $\bar{\phi_{1}}$ values are focused on, respectively. In Figure 14, there is a slight phase difference in the positive *C_V1_* peak in the downstroke. On the whole, the positive *C_V1_* and *C_H1_* peaks first increase and then decrease with the increase in $\bar{\phi_{1}}$, which is larger than that of the SW. The maximum force peaks are obtained at $\bar{\phi_{1}}$ = 25° (see the solid red line in Figure 14). In the upstroke, the force does not change significantly as $\bar{\phi_{1}}$ increases. This results in the vertical and horizontal force increments of the FW being largest at $\bar{\phi_{1}}$ = 25° (Figure 13a,b).

In Figure 15, the positive *C_V2_* and *C_H2_* peaks decrease as $\bar{\phi_{1}}$ increases and are smaller than those of the SW in the downstroke. In the upstroke, the negative *C_V2_* and positive *C_H2_* peaks increase with the increase in $\bar{\phi_{1}}$ and are larger than those of the SW. This results in a decrease in ∆$\bar{C_{V2}}$ and a slight increase in ∆$\bar{C_{H2}}$ as $\bar{\phi_{1}}$ increases (Figure 13c,d). Notably, there is also a phase difference between the changes in the negative *C_V2_* and positive *C_H2_* peaks in the upstroke. This is due to differences in the timing of the wake effect, which will be explained in more detail later.

**Figure 16:**
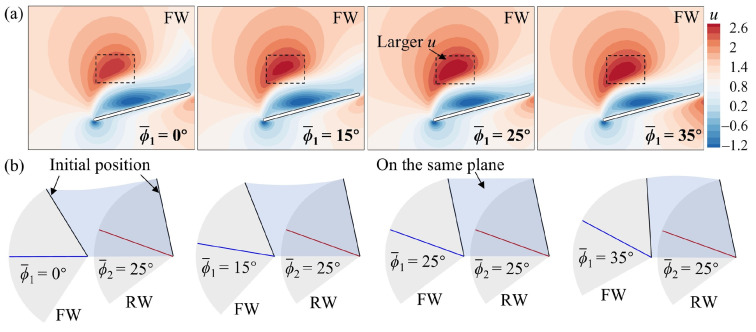
(**a**) Velocity (*u*) contour at the *r*_2_ plane of the FW for different $\bar{\phi_{1}}$ values at *L* = 1.5*c* and *t*/*T* = 0.25. *u* is the velocity vector component that is parallel to the stroke plane and perpendicular to the wingspan. (**b**) Schematic diagram of the flapping mean angles of (blue line) the FW and (red line) the RW.

To further elucidate the effect of $\bar{\phi_{1}}$ on the transient forces of the FW and the RW, a detailed flow analysis is performed. At *L* = 1.5*c*, the narrow channel effect similarly makes the aerodynamic forces of the FW higher than those of the SW in the downstroke (Figure 14). However, there are differences in the impact of the narrow channel effect at different $\bar{\phi_{1}}$ values, as shown in Figure 16. In Figure 16a, the relative incoming velocity (*u*) of the FW first increases and then decreases with the increase in $\bar{\phi_{1}}$, reaching a maximum at $\bar{\phi_{1}}$ = 25°. This leads to a change in the aerodynamic force of the FW (Figure 14). The variation in $\bar{\phi_{1}}$ inherently changes the initial positions of the FW and the RW, as shown in Figure 16b. It can be noticed from the figure that the initial positions of the FW and the RW are on the same plane when $\bar{\phi_{1}}$ = 25°, while the initial positions of the FW are lower and higher than those of the RW for $\bar{\phi_{1}}$ < 25° and $\bar{\phi_{1}}$ > 25°, respectively. If both wings move down from the same position, each wing will encounter a larger effective flow, which is the superposition of its own velocity and the induced flow due to the other wing [31]. In contrast, if there is a deviation in the initial position of the two wings, it reduces the effective incoming flow. This explains the difference in the relative incoming velocity of the FW due to the change in $\bar{\phi_{1}}$ and emphasizes that the initial positions of adjacent wings are on the same plane to maximize the impact of the narrow channel effect.

**Figure 17:**
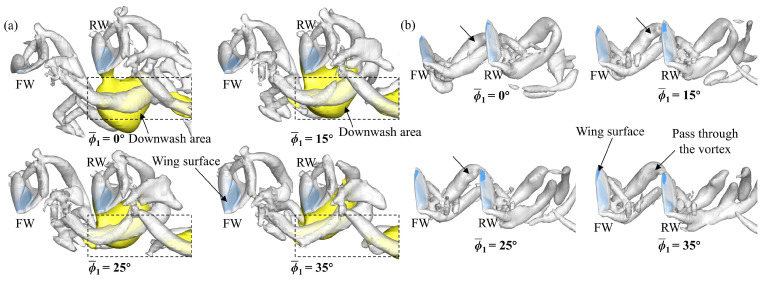
Iso-Q surface (Q = 0.2, gray) showing the vortex structures and iso-vertical velocity (*v*) surface (*v* = −0.4, yellow) showing the downwash for different $\bar{\phi_{1}}$ values at *L* = 5*c* in the (**a**) downstroke (*t*/*T* = 0.25) and (**b**) upstroke (*t*/*T* = 0.75).

At *L* = 5*c*, the narrow channel effect hardly plays a role in the downstroke, but the downwash effect reduces the forces of the RW (Figure 15). The position of the downwash area varies with different $\bar{\phi_{1}}$ values. In Figure 17a, the downwash area moves closer to the RW in the vertical direction as $\bar{\phi_{1}}$ increases. The closer the downwash area is, the stronger the downwash effect becomes. Therefore, the aerodynamic forces of the RW decrease as $\bar{\phi_{1}}$ increases. In the upstroke, the RW can capture the wake of the FW to increase aerodynamic forces (Figure 15). However, the strength and timing of the wake capture effect are different as $\bar{\phi_{1}}$ increases. In Figure 17b, the position of the shedding vortex of the FW relative to the RW gradually lifts (as indicated by the arrows) as $\bar{\phi_{1}}$ increases. This results in different positions and times of passing through the wake of the FW when the RW moves upward and forward. The larger the $\bar{\phi_{1}}$, the more wake the RW can capture, but the later the timing will be. This explains the change in the forces of the RW in the upstroke (Figure 15).

In summary, the change in $\bar{\phi_{1}}$ inherently alters the initial positions of the FW. At a smaller spacing, the narrow channel effect works better when the FW and the RW move downward from the same position. At a larger spacing, the impact of the downwash effect can be attenuated in the downstroke at $\bar{\phi_{1}}$ < 25°, and the impact of the wake capture effect can be strengthened in the upstroke at $\bar{\phi_{1}}$ > 25°.

### 3.3. Effect of Δtr_1_

**Figure 18:**
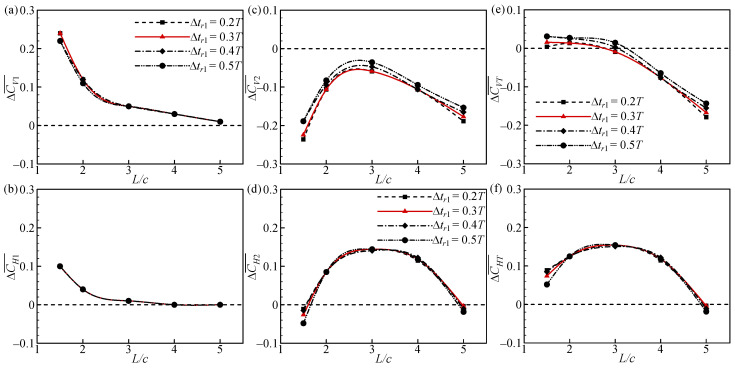
Increments of $\bar{C_{V}}$ and $\bar{C_{H}}$ of the (**a**,**b**) forewing, (**c**,**d**) hindwing, and (**e**,**f**) total tandem flapping wings relative to a single flapping wing at different Δ*tr_1_* and *L* values.

Figure 18 shows the increments of $\bar{C_{V}}$ and $\bar{C_{H}}$ for the forewing, hindwing, and overall tandem flapping wings relative to a single flapping wing at different Δ*tr*_1_ and *L* values. In Figure 18a,b, ∆$\bar{C_{V1}}$ and ∆$\bar{C_{H1}}$ decrease with increasing *L*, but they are almost constant as Δ*tr*_1_ changes. ∆$\bar{C_{V2}}$ and ∆$\bar{C_{H2}}$ increase and then decrease as *L* increases, and the change in Δ*tr*_1_ has a slight effect on ∆$\bar{C_{V2}}$ but not on ∆$\bar{C_{H2}}$ (Figure 18c,d). As a result, ∆$\bar{C_{VT}}$ and ∆$\bar{C_{\mathrm{HT}}}$ are mainly affected by *L*, and to a lesser extent by Δ*tr*_1_ (Figure 18e,f). This indicates that the aerodynamic forces of tandem flapping wings are not significantly affected by modifying Δ*tr*_1_ at different wing spacings.

## 4. Conclusions

This study focused on the effect of forewing kinematics on the unsteady aerodynamic forces of three-dimensional tandem flapping wings through numerical simulations. The kinematic parameters of the hindwing remained unchanged, while those of the forewing were modified. The forewing kinematics parameters include flapping amplitude (*Φ*_1_), flapping mean angle ($\bar{\phi_{1}}$), and pitch rotation duration (Δ*tr*_1_). In addition, the effect of wing spacing (*L*) is also considered. The results show that adjusting *Φ*_1_ and $\bar{\phi_{1}}$ significantly affects the aerodynamic forces, while Δ*tr*_1_ has little effect. More importantly, the aerodynamic effects of *Φ*_1_ and $\bar{\phi_{1}}$ are distinct at different *L* values. The flapping amplitude of the forewing is smaller than that of the hindwing by adjusting *Φ*_1_, which is beneficial for total aerodynamic force generation at a small *L*, and the opposite is true at a large *L*. The modification of $\bar{\phi_{1}}$ essentially changes the initial position of the forewing. A small $\bar{\phi_{1}}$ makes the forewing below the hindwing, which is detrimental to total vertical forces at a small *L*, but the effect is reversed at a large *L*. This suggests that optimal aerodynamic forces can be maintained at different wing spacings by adjusting forewing kinematics. The aerodynamic force generation of tandem wings is strongly related to wing–wing interactions. Flow analyses indicate that the variations in *Φ*_1_ and $\bar{\phi_{1}}$ would primarily change the extent of the influence of the narrow channel effect, the downwash effect, and the wake capture effect that have been proposed. With a large *Φ*_1_, the narrow channel effect in the downstroke and the wake capture effect in the upstroke are enhanced, and the downwash effect in the downstroke is weakened. With a large $\bar{\phi_{1}}$, the wake capture effect in the upstroke is strengthened, and the downwash effect in the downstroke is attenuated with a small $\bar{\phi_{1}}$. Additionally, the narrow channel effect works best when the forewing and hindwing maintain the same flapping mean angle. Thus, the flapping amplitude and flapping mean angle of the forewing can be artificially controlled to improve the aerodynamic performance in the design of tandem flapping wing micro-air vehicles.

## Figures and Tables

**Table 1:** Parameters of the forewing that were examined.

Flapping Amplitude (*Φ*_1_)	Flapping Mean Angle ($\bar{\phi_{1}}$)	Pitch Rotation Duration (Δ*tr*_1_)	Wing Spacing (*L*)
60°, 80°, 100°, 120°	25°	0.3*T*	1.5*c*, 2*c*, 3*c*, 4*c*, 5*c*
100°	0°, 5°, 15°, 25°, 35°	0.3*T*	
100°	25°	0.2*T*, 0.3*T*, 0.4*T*, 0.5*T*	

**Figure A1:**
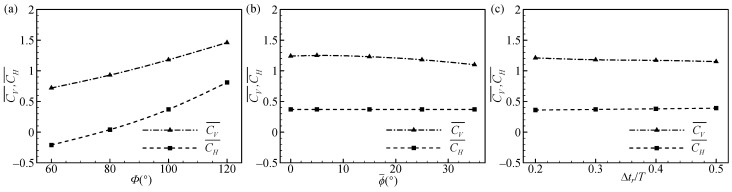
Cycle-averaged vertical ($\bar{C_{V}}$) and horizontal ($\bar{C_{H}}$) force coefficients of the single flapping wing at different (**a**) *Φ*, (**b**) $\overset{-}{\phi }$, and (**c**) Δ*tr* values.

## Data Availability

The data that support the findings of this study are available from the corresponding author upon reasonable request.

## Appendix A

The period of flapping cycles of the single flapping wing is always the same as that of the hindwing at different parameters. Cycle-averaged vertical ($\bar{C_{V}}$) and horizontal ($\bar{C_{H}}$) force coefficients are calculated for the single flapping wing at different *Φ*, $\overset{-}{\phi }$, and Δ*tr* values, as shown in Figure A1.
